# Supplementary figures and images for: Transmission of SARS-CoV-2 in domestic cats imposes a narrow bottleneck
Source: PLoS Pathog. 2021 Feb 26;17(2):e1009373. doi: 10.1371/journal.ppat.1009373 (PMC7946358; doi:10.1371/journal.ppat.1009373)

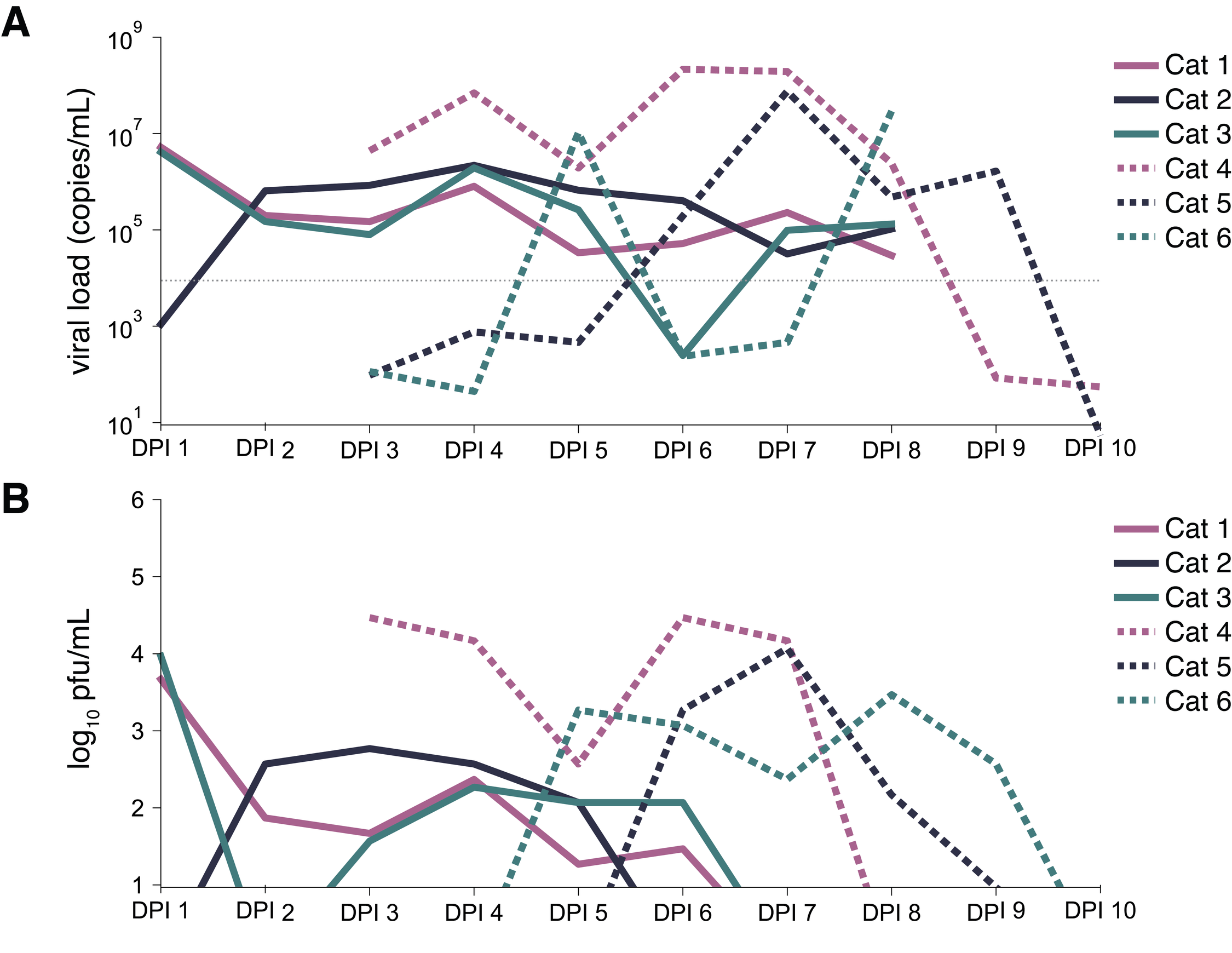

Supplement: S1 Fig — A) Viral RNA burden over time for each cat. Index cats are represented by a solid line and contact cats are represented by a dashed line. Transmission pairs are denoted by color. The grey, horizontal dotted line represents when less than ~100 copies/μL are input into the reverse transcription reaction. B) Infectious viral titer over time. Index cats are represented by a solid line and contact cats are represented by a dashed line. Transmission pairs are denoted by color. (TIF) [file ppat.1009373.s001.tif]

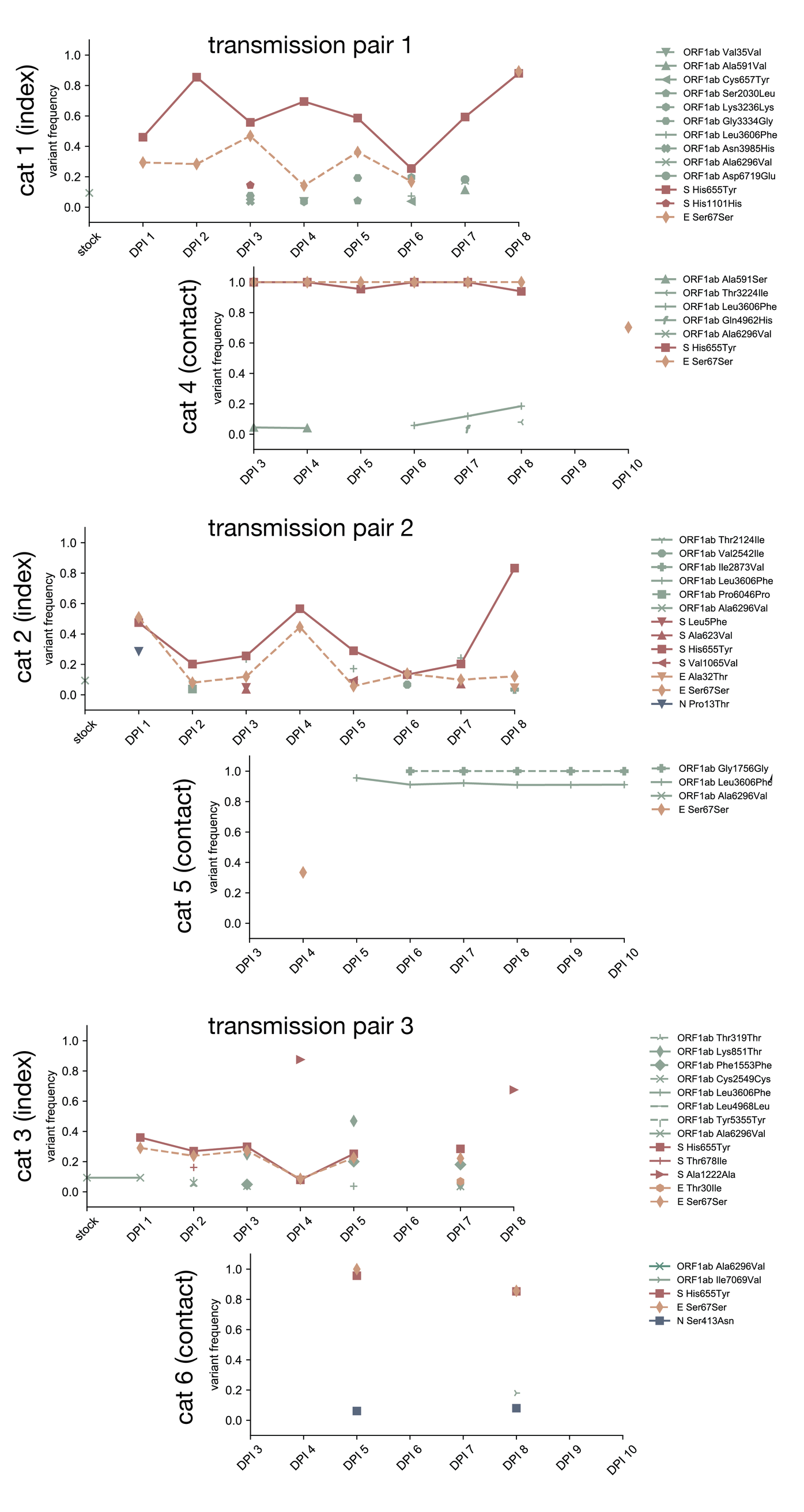

Supplement: S2 Fig — Each variant is colored based on gene location. Nonsynonymous variants are plotted with solid lines and synonymous variants are plotted with dashed lines. Days with viral loads too low to yield high quality sequences are shown by the gaps in data (i.e. cat 3 day 6 and cat 4 day 9). (TIF) [file ppat.1009373.s002.tif]

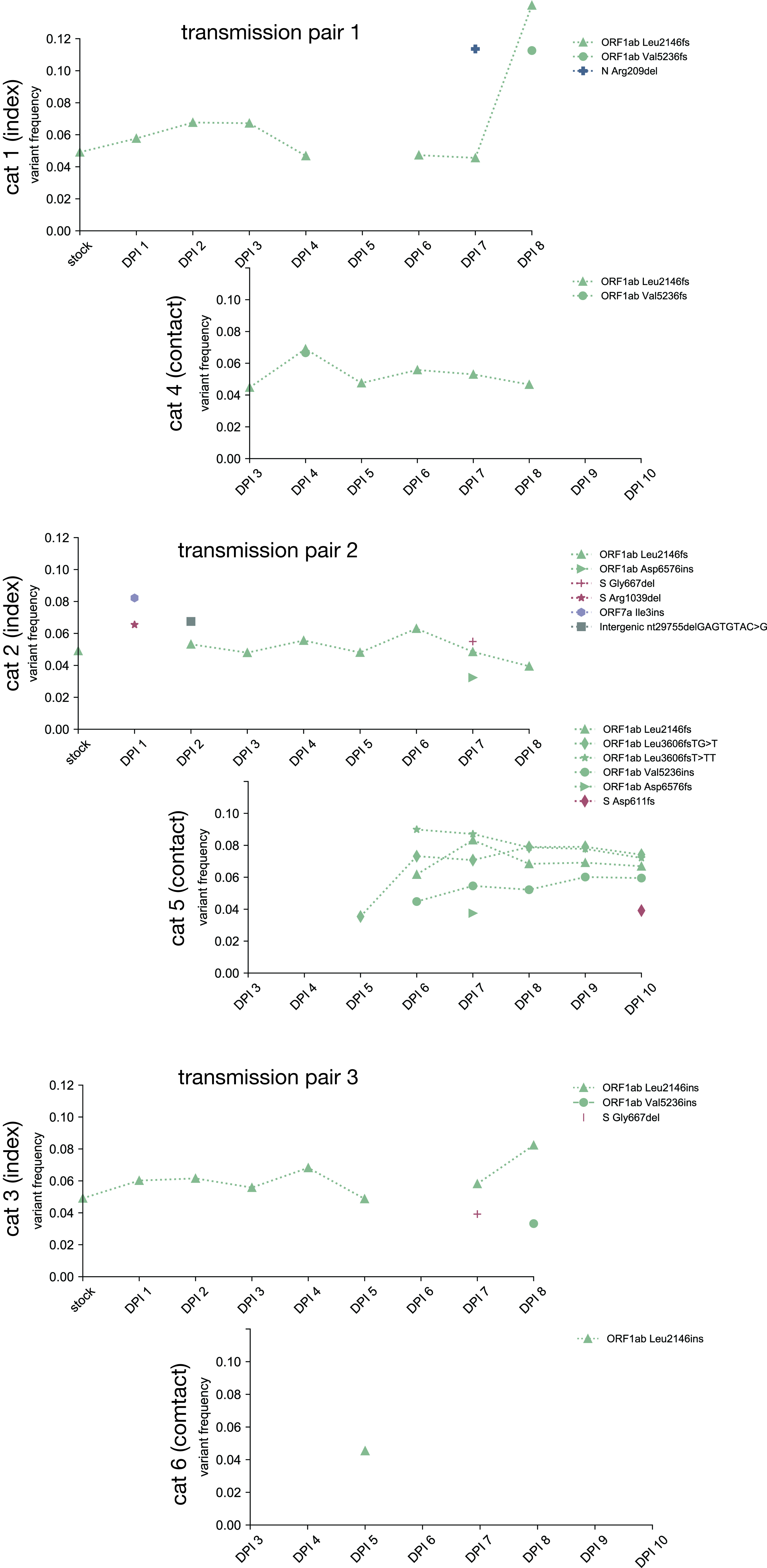

Supplement: S3 Fig — Each indel is colored based on gene location. Days with viral loads too low to yield high quality sequences are shown by the gaps in data (i.e. cat 3 day 6 and cat 4 day 9). Note the y-axis range is 0–12%, not 0–100%, to facilitate readability. (TIF) [file ppat.1009373.s003.tif]

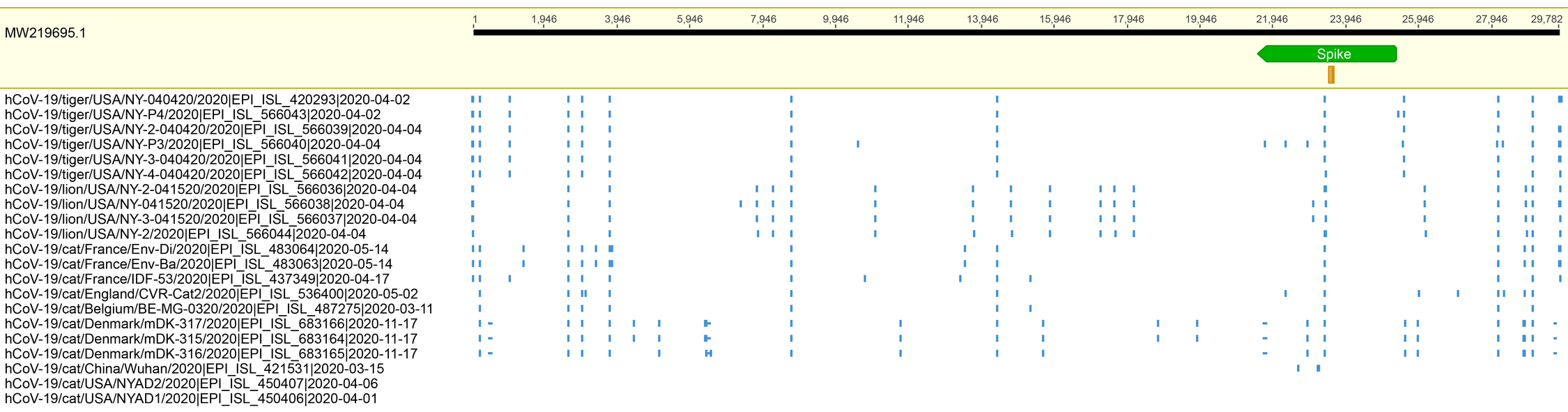

Supplement: S4 Fig — Sequences were aligned against MW219695.1, the inoculum virus used in these experiments. Consensus-level differences are highlighted with a blue vertical marker. Indels are noted with a horizontal vertical marker. The spike open reading frame is annotated with a green marker and site amino acid 655 in Spike is highlighted with the orange box. None of these sequences contain a consensus mutation at residue 655 in Spike. (TIF) [file ppat.1009373.s004.tif]

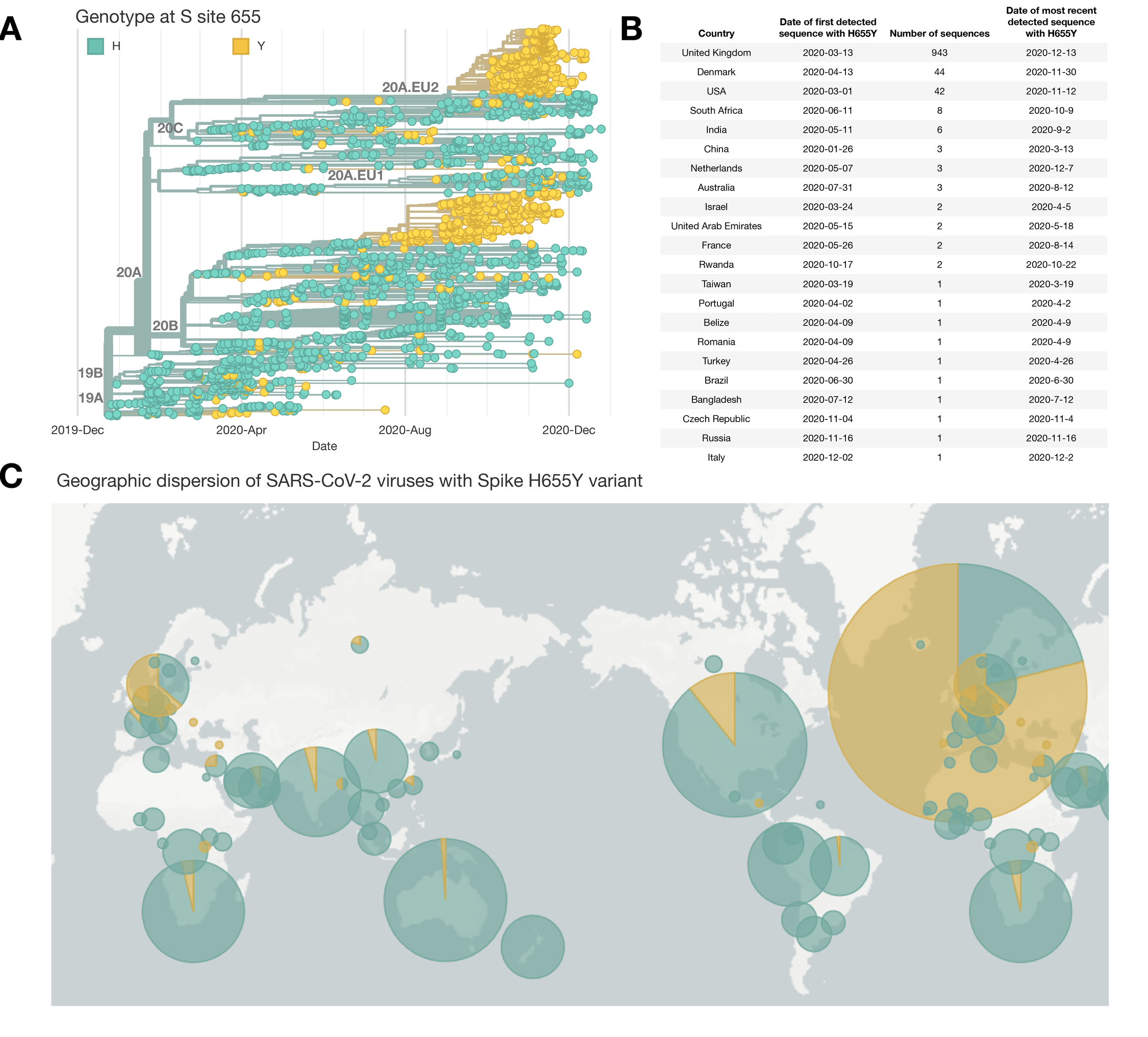

Supplement: S5 Fig — A) A time-resolved phylogeny focused on viruses that contain Spike H655Y. Viruses that contain histidine (H) at Spike 655 are colored in teal. Viruses with tyrosine (Y) at Spike 655 are colored in yellow. B) Counts of SARS-CoV-2 viruses that contain Spike H655Y, broken down by country. C) Map highlighting the number viruses from each country. The size of the circle represents the number of sequences from the appropriate country contained in the phylogeny. (TIF) [file ppat.1009373.s005.tif]

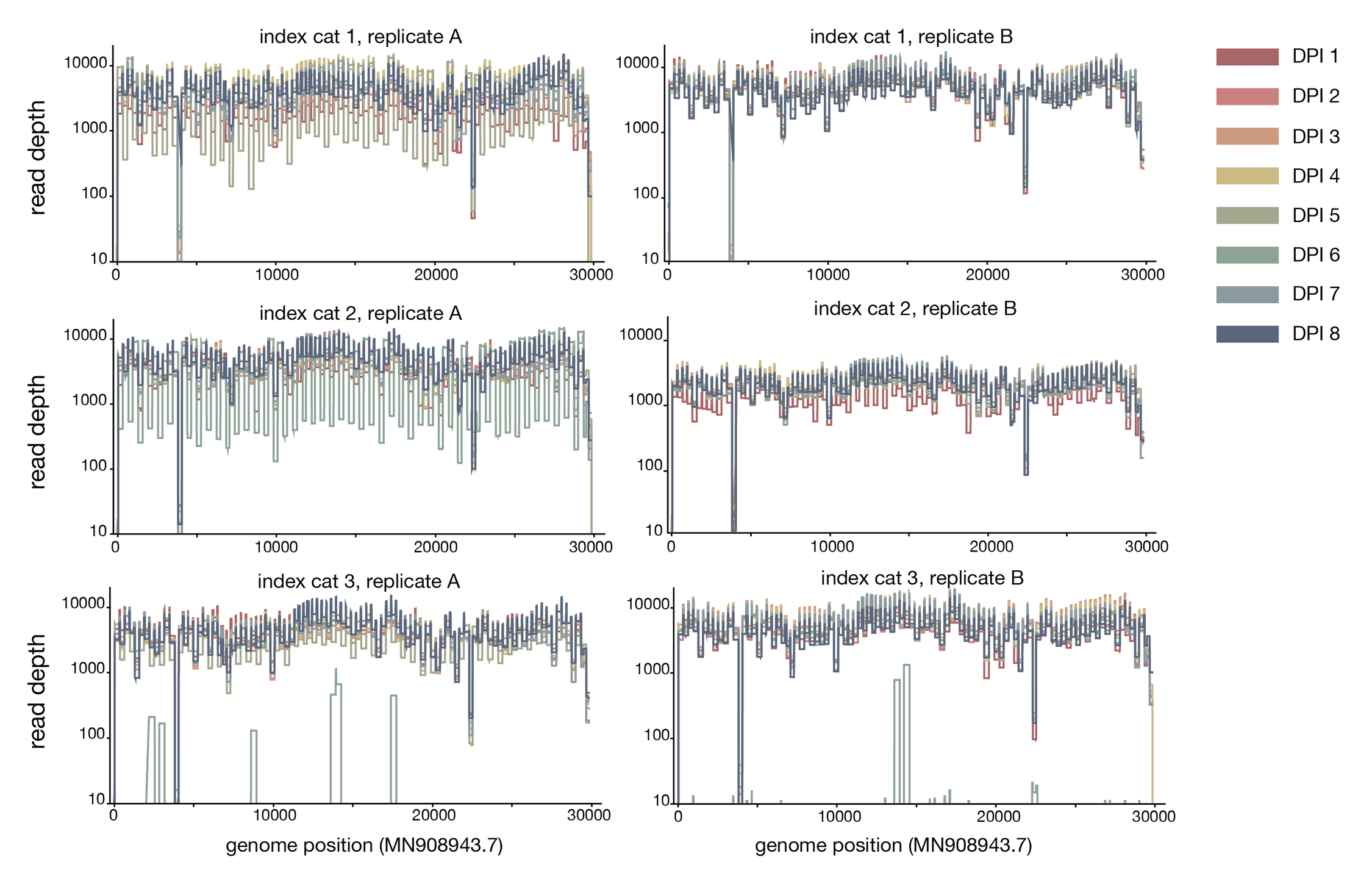

Supplement: S6 Fig — Each day is represented by a different color. Replicate A is shown in the left column and replicate B is shown in the right column. (TIF) [file ppat.1009373.s006.tif]

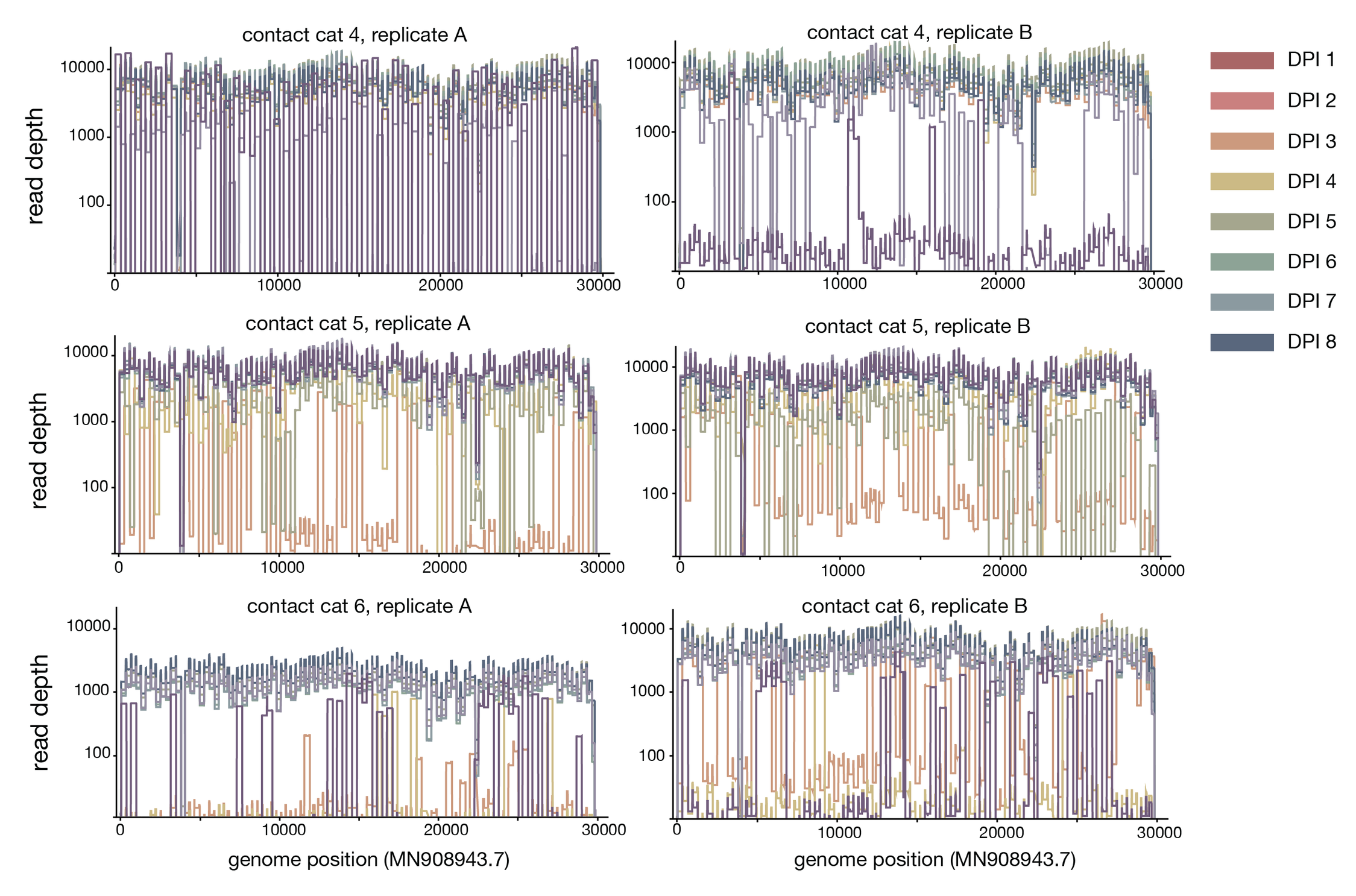

Supplement: S7 Fig — Each day is represented by a different color. Replicate A is shown in the left column and replicate B is shown in the right column. (TIF) [file ppat.1009373.s007.tif]

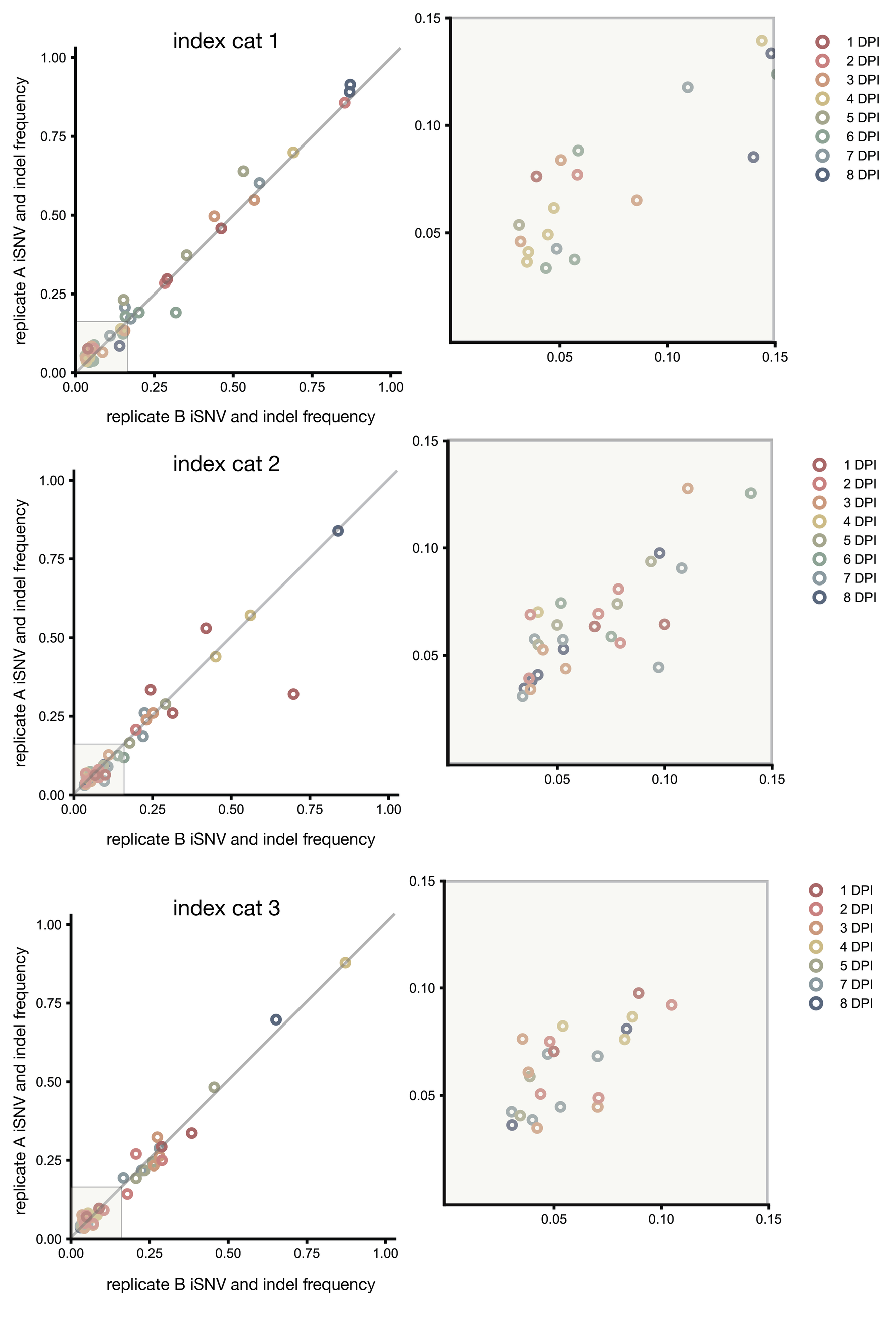

Supplement: S8 Fig — The frequency of each variant per replicate is shown here. The diagonal line represents the 1:1 intersection of replicate variants. The subplot to the right of each primary plot is a zoomed-in view of the low-frequency variants (3–15%). Each timepoint is denoted by a different color. (TIF) [file ppat.1009373.s008.tif]

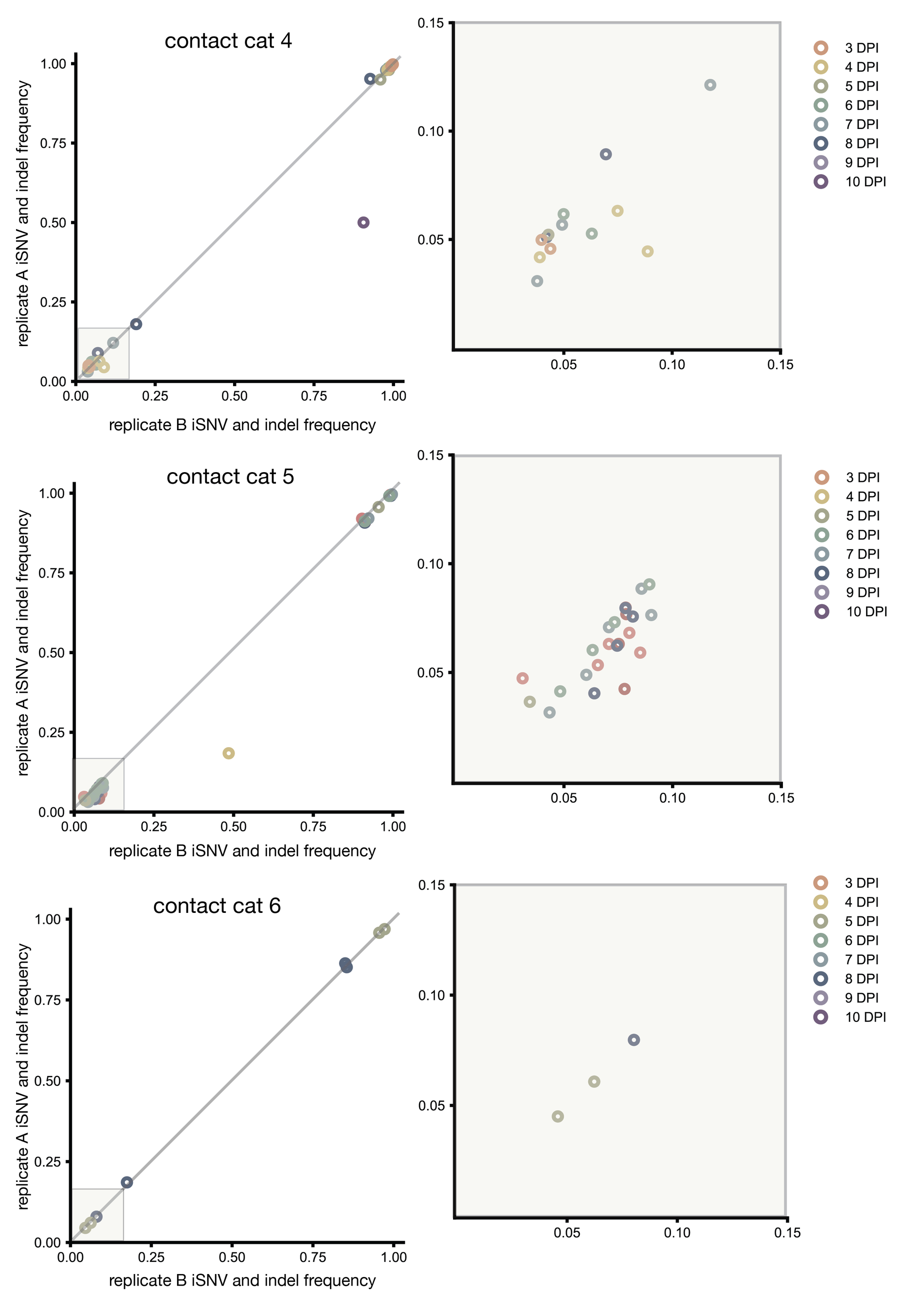

Supplement: S9 Fig — The frequency of each variant per replicate is shown here. The diagonal line represents the 1:1 intersection of replicate variants. The subplot to the right of each primary plot is a zoomed-in view of the low-frequency variants (3–15%). Each timepoint is denoted by a different color. (TIF) [file ppat.1009373.s009.tif]

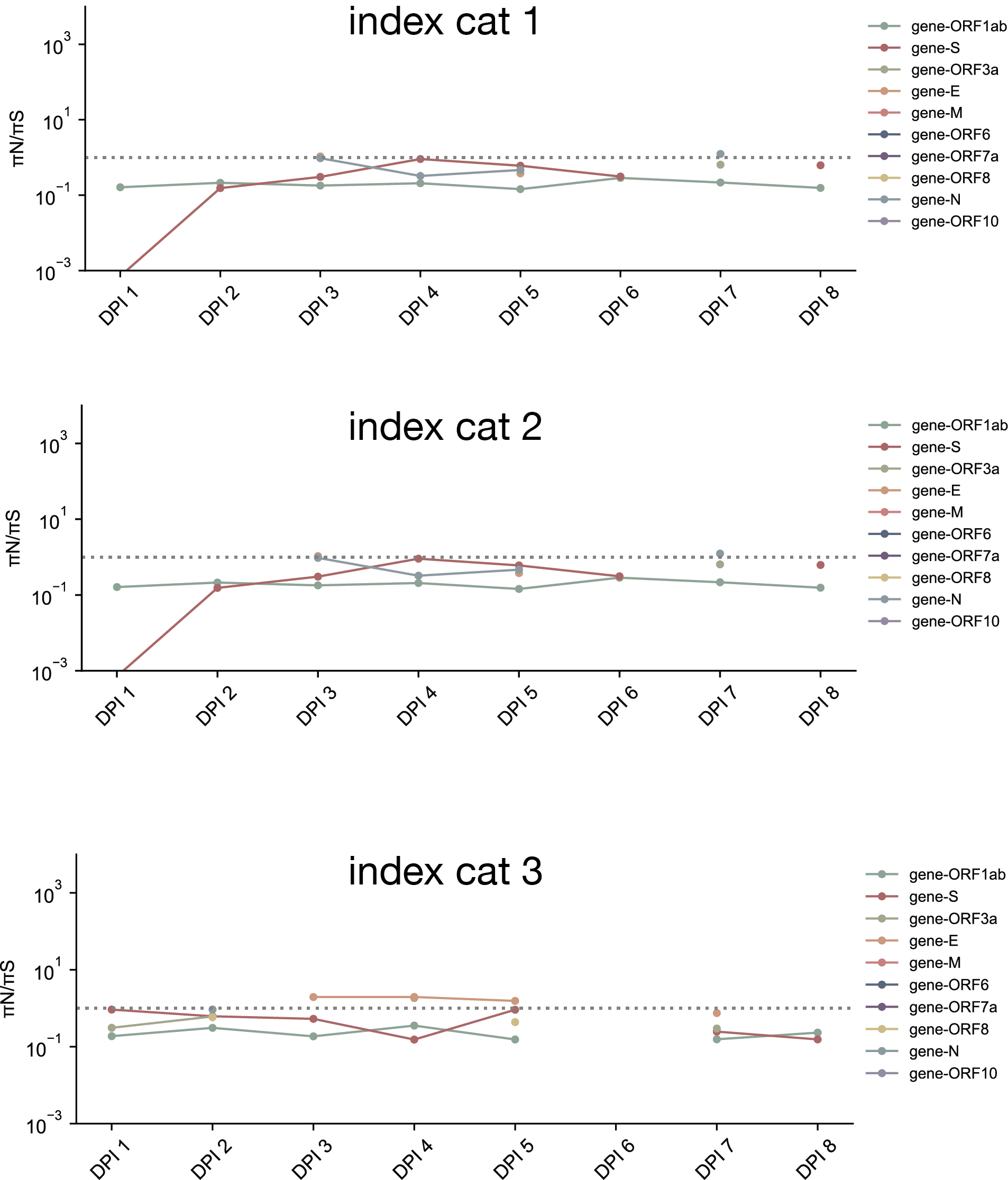

Supplement: S10 Fig — Line color denotes gene. The horizontal dotted gray line is plotted at y = 1 or when πN ~ πS. (TIF) [file ppat.1009373.s010.tif]

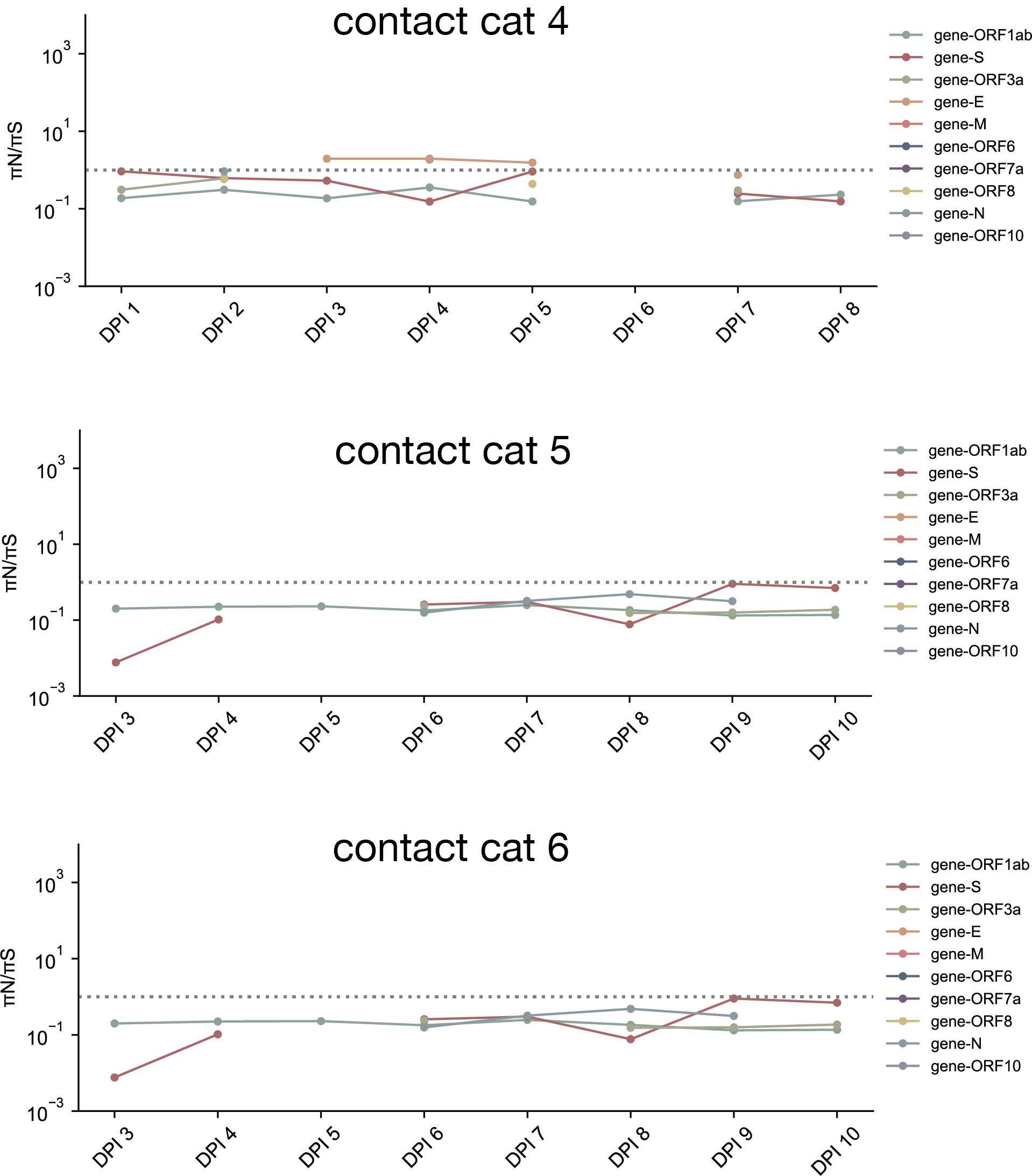

Supplement: S11 Fig — Line color denotes gene. The horizontal dotted gray line is plotted at y = 1 or when πN ~ πS. (TIF) [file ppat.1009373.s011.tif]

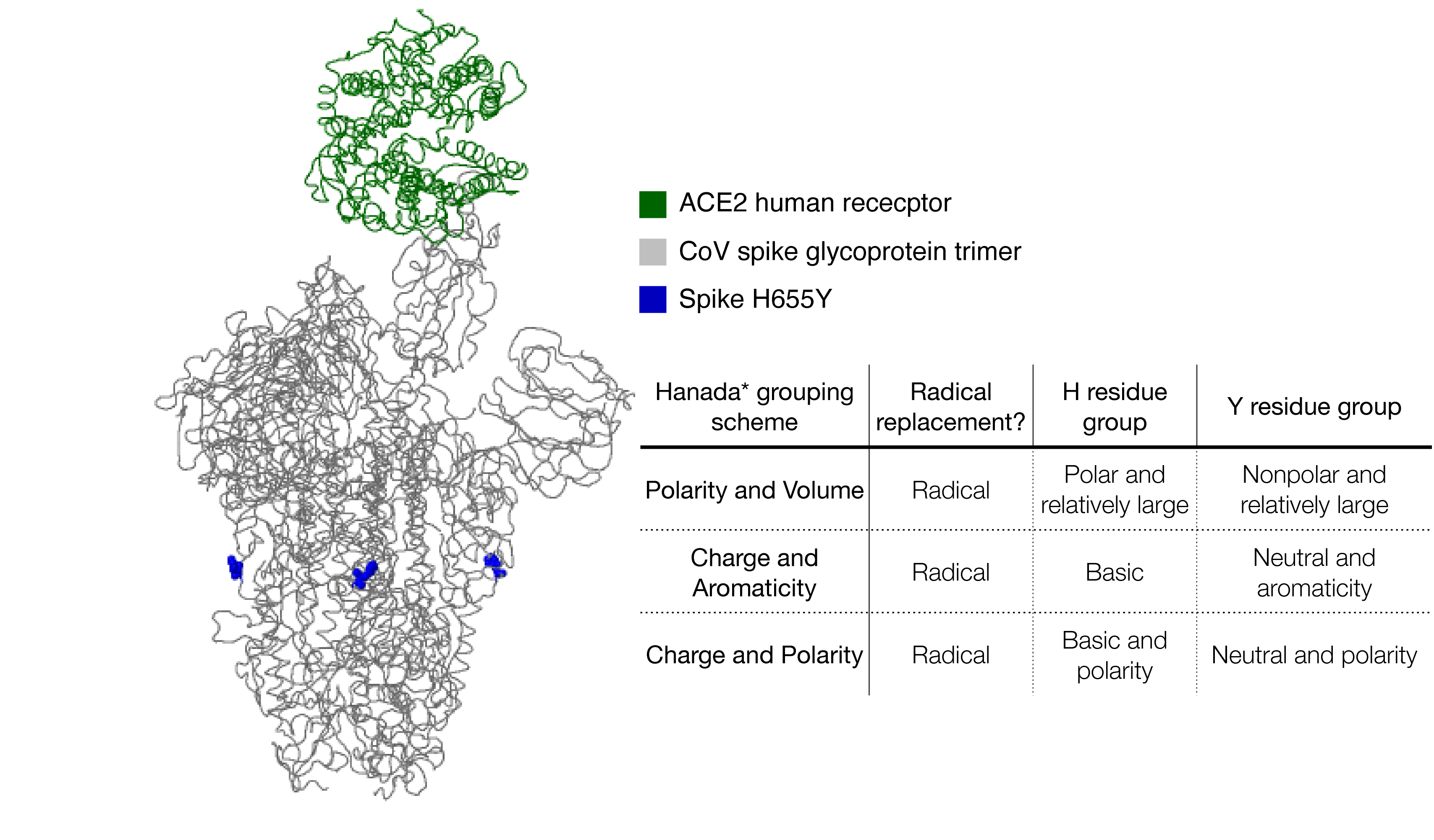

Supplement: S12 Fig — Spike H655Y is highlighted in blue. The table to the right of the crystal structure includes summary information regarding the impact of a histidine to tyrosine change on amino acid charge, volume, and aromaticity. * Qualitative definitions of radical amino acid replacements, based on three alternative residue groupings, see Hanada et al., 2006 [68]. The crystal structure and summary information were generated using GISAID’s CoVserver mutation analysis tool. (TIF) [file ppat.1009373.s012.tif]
