# Supplementary material for: Transmission of SARS-CoV-2 in domestic cats imposes a narrow bottleneck
Source: PLoS Pathog. 2021 Feb 26;17(2):e1009373. doi: 10.1371/journal.ppat.1009373 (PMC7946358; doi:10.1371/journal.ppat.1009373)
Supplement: S1 Table — (PDF) [file ppat.1009373.s013.pdf]

	mean πS	std πS	mean πN	std πN	πN/πS	statistic, p-value
index cat 1						
**ORF1ab**	0.015670	0.005609	0.003019	0.001124	0.195265	(statistic=6.23, pvalue=2.11e-05)
**S**	0.001564	0.000600	0.000644	0.000467	0.413995	(statistic=3.34, pvalue=0.005)
**ORF3a**	0.005367	0.000001	0.005878	0.002267	0.641899	(statistic=-0.303, pvalue=0.772)
**E**	0.011707	0.010139	0.011930	0.005719	0.719601	(statistic=-0.0456, pvalue=0.965)
**M**			0.002138	0.000059		(statistic=nan, pvalue=nan)
**ORF6**			0.007395			(statistic=nan, pvalue=nan)
**ORF7a**	0.011992	0.000487				(statistic=nan, pvalue=nan)
**ORF8**	0.031186	0.012756				(statistic=nan, pvalue=nan)
**N**	0.005292	0.002941	0.003036	0.001403	0.744626	(statistic=1.410, pvalue=0.196)
**ORF10**						(statistic=nan, pvalue=nan)
index cat 2						
**ORF1ab**	0.025570	0.007229	0.005423	0.001061	0.219071	(statistic=7.799, pvalue=1.837e-06)
**S**	0.004651	0.002567	0.001476	0.000556	0.457442	(statistic=3.419, pvalue=0.0042)
**ORF3a**	0.008660	0.003052	0.003867	0.001630	0.535879	(statistic=3.549, pvalue=0.0053)
**E**	0.008410	0.009696	0.015842	0.010396	436.372255	(statistic=-1.191, pvalue=0.261)
**M**			0.002479	0.000841		(statistic=nan, pvalue=nan)
**ORF6**			0.007468			(statistic=nan, pvalue=nan)
**ORF7a**	0.011872					(statistic=nan, pvalue=nan)
**ORF8**	0.030343	0.012115	0.005673	0.002620	0.224566	(statistic=2.733, pvalue=0.0292)
**N**	0.006988	0.004962	0.001398	0.000535	0.346156	(statistic=2.240, pvalue=0.066)
**ORF10**						(statistic=nan, pvalue=nan)
index cat 3						
**ORF1ab**	0.022195	0.005336	0.004752	0.000970	0.225357	(statistic=8.509, pvalue=1.988e-06)
**S**	0.003619	0.001697	0.001584	0.001032	0.505292	(statistic=2.711, pvalue=0.0189)
**ORF3a**	0.005516	0.000037	0.004351	0.003509	0.764893	(statistic=0.649, pvalue=0.533)
**E**	0.016174	0.000532	0.023985	0.007123	1.553581	(statistic=-2.145, pvalue=0.0642)
**M**	0.006492		0.002078	0.000007		(statistic=nan, pvalue=nan)
**ORF6**						(statistic=nan, pvalue=nan)
**ORF7a**	0.012294	0.000305				(statistic=nan, pvalue=nan)
**ORF8**	0.020154	0.006891	0.008740	0.002221	0.509847	(statistic=2.708, pvalue=0.0352)
**N**	0.005320	0.001974	0.003336		0.925883	(statistic=nan, pvalue=nan)
**ORF10**						(statistic=nan, pvalue=nan)
