# Supplementary material for: Transmission of SARS-CoV-2 in domestic cats imposes a narrow bottleneck
Source: PLoS Pathog. 2021 Feb 26;17(2):e1009373. doi: 10.1371/journal.ppat.1009373 (PMC7946358; doi:10.1371/journal.ppat.1009373)
Supplement: S2 Table — (PDF) [file ppat.1009373.s014.pdf]

	Cat 1	Cat 2	Cat 3	Cat 4	Cat 5	Cat 6
**π DPI 1**	0.000246	0.000290	0.000433			
**π DPI 2**	0.000314	0.000705	0.000546			
**π DPI 3**	0.000458	0.000557	0.000781	0.000712	0.000153	
**π DPI 4**	0.000577	0.000650	0.000568	0.000796	0.000206	0.000037
**π DPI 5**	0.000489	0.000513	0.000540	0.001007	0.000149	0.000576
**π DPI 6**	0.000430	0.000720		0.000917	0.000854	0.000156
**π DPI 7**	0.000365	0.000541	0.000683	0.000721	0.000876	0.000025
**π DPI 8**	0.000214	0.000591	0.000458	0.000879	0.000872	0.000720
**π DPI 9**				0.000125	0.000965	0.000070
**π DPI 10**				0.000371	0.000932	0.000000
**mean π**	0.000387	0.000571	0.000573	0.000691	0.000626	0.000226
**std π**	0.000117	0.000128	0.000114	0.000279	0.000355	0.000273
