# Supplementary material for: Transmission of SARS-CoV-2 in domestic cats imposes a narrow bottleneck
Source: PLoS Pathog. 2021 Feb 26;17(2):e1009373. doi: 10.1371/journal.ppat.1009373 (PMC7946358; doi:10.1371/journal.ppat.1009373)
Supplement: S3 Table — (PDF) [file ppat.1009373.s015.pdf]

name	pool	sequence	length	%gc	tm (use 65)
nCoV-2019_1_LEFT	nCoV-2019_1	ACCAACCAACTTTCGATCTCTTGT	24	41.67	60.69
nCoV-2019_1_RIGHT	nCoV-2019_1	CATCTTTAAGATGTTGACGTGCCTC	25	44	60.45
nCoV-2019_2_LEFT	nCoV-2019_2	CTGTTTTACAGGTTCGCGACGT	22	50	61.67
nCoV-2019_2_RIGHT	nCoV-2019_2	TAAGGATCAGTGCCAAGCTCGT	22	50	61.74
nCoV-2019_3_LEFT	nCoV-2019_1	CGGTAATAAAGGAGCTGGTGGC	22	54.55	61.32
nCoV-2019_3_RIGHT	nCoV-2019_1	AAGGTGTCTGCAATTCATAGCTCT	24	41.67	60.32
nCoV-2019_4_LEFT	nCoV-2019_2	GGTGTATACTGCTGCCGTGAAC	22	54.55	61.56
nCoV-2019_4_RIGHT	nCoV-2019_2	CACAAGTAGTGGCACCTTCTTTAGT	25	44	60.97
nCoV-2019_5_LEFT	nCoV-2019_1	TGGTGAAACTTCATGGCAGACG	22	50	61.39
nCoV-2019_5_RIGHT	nCoV-2019_1	ATTGATGTTGACTTTCTCTTTTTGGAGT	28	32.14	60.17
nCoV-2019_6_LEFT	nCoV-2019_2	GGTGTTGTTGGAGAAGGTTCCG	22	54.55	61.64
nCoV-2019_6_RIGHT	nCoV-2019_2	TAGCGGCCTTCTGTAAAACACG	22	50	61.18
nCoV-2019_7_LEFT	nCoV-2019_1	ATCAGAGGCTGCTCGTGTTGTA	22	50	61.73
nCoV-2019_7_LEFT_alt0	nCoV-2019_1	CATTTGCATCAGAGGCTGCTCG	22	54.55	62.44
nCoV-2019_7_RIGHT	nCoV-2019_1	TGCACAGGTGACAATTTGTCCA	22	45.45	60.95
nCoV-2019_7_RIGHT_alt5	nCoV-2019_1	AGGTGACAATTTGTCCACCGAC	22	50	61.07
nCoV-2019_8_LEFT	nCoV-2019_2	AGAGTTTCTTAGAGACGGTTGGGA	24	45.83	61
nCoV-2019_8_RIGHT	nCoV-2019_2	GCTTCAACAGCTTCACTAGTAGGT	24	45.83	60.56
nCoV-2019_9_LEFT	nCoV-2019_1	TCCCACAGAAGTGTTAACAGAGGA	24	45.83	61.18
nCoV-2019_9_LEFT_alt4	nCoV-2019_1	TTCCCACAGAAGTGTTAACAGAGG	24	45.83	60.44
nCoV-2019_9_RIGHT	nCoV-2019_1	ATGACAGCATCTGCCACAACAC	22	50	61.71
nCoV-2019_9_RIGHT_alt2	nCoV-2019_1	GACAGCATCTGCCACAACACAG	22	54.55	62.26
nCoV-2019_10_LEFT	nCoV-2019_2	TGAGAAGTGCTCTGCCTATACAGT	24	45.83	61.12
nCoV-2019_10_RIGHT	nCoV-2019_2	TCATCTAACCAATCTTCTTCTTGCTCT	27	37.04	60.31
nCoV-2019_11_LEFT	nCoV-2019_1	GGAATTTGGTGCCACTTCTGCT	22	50	61.66
nCoV-2019_11_RIGHT	nCoV-2019_1	TCATCAGATTCAACTTGCATGGCA	24	41.67	61.35
nCoV-2019_12_LEFT	nCoV-2019_2	AAACATGGAGGAGGTGTTGCAG	22	50	61.08
nCoV-2019_12_RIGHT	nCoV-2019_2	TTCACTCTTCATTTCCAAAAAGCTTGA	27	33.33	60.36
nCoV-2019_13_LEFT	nCoV-2019_1	TCGCACAAATGTCTACTTAGCTGT	24	41.67	60.56
nCoV-2019_13_RIGHT	nCoV-2019_1	ACCACAGCAGTTAAAACACCCT	22	45.45	60.36
nCoV-2019_14_LEFT	nCoV-2019_2	CATCCAGATTCTGCCACTCTTGT	23	47.83	60.62

nCoV-2019_14_LEFT_alt4	nCoV-2019_2	TGGCAATCTTCATCCAGATTCTGC	24	45.83	61.47
nCoV-2019_14_RIGHT	nCoV-2019_2	AGTTTCCACACAGACAGGCATT	22	45.45	60.42
nCoV-2019_14_RIGHT_alt2	nCoV-2019_2	TGCGTGTTTCTTCTGCATGTGC	22	50	62.76
nCoV-2019_15_LEFT	nCoV-2019_1	ACAGTGCTTAAAAAGTGTAAAAGTGCC	27	37.04	61.32
nCoV-2019_15_LEFT_alt1	nCoV-2019_1	AGTGCTTAAAAAGTGTAAAAGTGCCT	26	34.62	60.13
nCoV-2019_15_RIGHT	nCoV-2019_1	AACAGAAACTGTAGCTGGCACT	22	45.45	60.16
nCoV-2019_15_RIGHT_alt3	nCoV-2019_1	ACTGTAGCTGGCACTTTGAGAGA	23	47.83	61.57
nCoV-2019_16_LEFT	nCoV-2019_2	AATTTGGAAGAAGCTGCTCGGT	22	45.45	60.82
nCoV-2019_16_RIGHT	nCoV-2019_2	CACAACTTGCGTGTGGAGGTTA	22	50	61.32
nCoV-2019_17_LEFT	nCoV-2019_1	CTTCTTTCTTTGAGAGAAGTGAGGACT	27	40.74	60.69
nCoV-2019_17_RIGHT	nCoV-2019_1	TTTGTTGGAGTGTTAACAATGCAGT	25	36	60.11
nCoV-2019_18_LEFT	nCoV-2019_2	TGGAAATACCCACAAGTTAATGGTTTAAC	29	34.48	60.69
nCoV-2019_18_LEFT_alt2	nCoV-2019_2	ACTTCTATTAAATGGGCAGATAACAACTGT	30	33.33	61.38
nCoV-2019_18_RIGHT	nCoV-2019_2	AGCTTGTTTACCACACGTACAAGG	24	45.83	61.51
nCoV-2019_18_RIGHT_alt1	nCoV-2019_2	GCTTGTTTACCACACGTACAAGG	23	47.83	60.3
nCoV-2019_19_LEFT	nCoV-2019_1	GCTGTTATGTACATGGGCACACT	23	47.83	61.18
nCoV-2019_19_RIGHT	nCoV-2019_1	TGTCCAACTTAGGGTCAATTTCTGT	25	40	60.4
nCoV-2019_20_LEFT	nCoV-2019_2	ACAAAGAAAACAGTTACACAACAACCA	27	33.33	60.68
nCoV-2019_20_RIGHT	nCoV-2019_2	ACGTGGCTTTATTAGTTGCATTGTT	25	36	60.28
nCoV-2019_21_LEFT	nCoV-2019_1	TGGCTATTGATTATAAACACTACACACCC	29	37.93	61.49
nCoV-2019_21_LEFT_alt2	nCoV-2019_1	GGCTATTGATTATAAACACTACACACCCT	29	37.93	61.29
nCoV-2019_21_RIGHT	nCoV-2019_1	TAGATCTGTGTGGCCAACCTCT	22	50	60.83
nCoV-2019_21_RIGHT_alt0	nCoV-2019_1	GATCTGTGTGGCCAACCTCTTC	22	54.55	61.2
nCoV-2019_22_LEFT	nCoV-2019_2	ACTACCGAAGTTGTAGGAGACATTATACT	29	37.93	61.25
nCoV-2019_22_RIGHT	nCoV-2019_2	ACAGTATTCTTTGCTATAGTAGTCGGC	27	40.74	60.73
nCoV-2019_23_LEFT	nCoV-2019_1	ACAACTACTAACATAGTTACACGGTGT	27	37.04	60.26
nCoV-2019_23_RIGHT	nCoV-2019_1	ACCAGTACAGTAGGTTGCAATAGTG	25	44	60.57
nCoV-2019_24_LEFT	nCoV-2019_2	AGGCATGCCTTCTTACTGTACTG	23	47.83	60.37
nCoV-2019_24_RIGHT	nCoV-2019_2	ACATTCTAACCATAGCTGAAATCGGG	26	42.31	61.19
nCoV-2019_25_LEFT	nCoV-2019_1	GCAATTGTTTTTCAGCTATTTTGCAGT	27	33.33	60.73
nCoV-2019_25_RIGHT	nCoV-2019_1	ACTGTAGTGACAAGTCTCTCGCA	23	47.83	61.3
nCoV-2019_26_LEFT	nCoV-2019_2	TTGTGATACATTCTGTGCTGGTAGT	25	40	60.28

nCoV-2019_26_RIGHT	nCoV-2019_2	TCCGCACTATCACCAACATCAG	22	50	60.42
nCoV-2019_27_LEFT	nCoV-2019_1	ACTACAGTCAGCTTATGTGTCAACC	25	44	60.8
nCoV-2019_27_RIGHT	nCoV-2019_1	AATACAAGCACCAAGGTCACGG	22	50	61.13
nCoV-2019_28_LEFT	nCoV-2019_2	ACATAGAAGTTACTGGCGATAGTTGT	26	38.46	60.13
nCoV-2019_28_RIGHT	nCoV-2019_2	TGTTTAGACATGACATGAACAGGTGT	26	38.46	60.91
nCoV-2019_29_LEFT	nCoV-2019_1	ACTTGTGTTCCTTTTTGTTGCTGC	24	41.67	61.39
nCoV-2019_29_RIGHT	nCoV-2019_1	AGTGTACTCTATAAGTTTTGATGGTGTGT	29	34.48	60.69
nCoV-2019_30_LEFT	nCoV-2019_2	GCACAACTAATGGTGACTTTTTGCA	25	40	61.19
nCoV-2019_30_RIGHT	nCoV-2019_2	ACCACTAGTAGATACACAAACACCAG	26	42.31	60.3
nCoV-2019_31_LEFT	nCoV-2019_1	TTCTGAGTACTGTAGGCACGGC	22	54.55	62.03
nCoV-2019_31_RIGHT	nCoV-2019_1	ACAGAATAAACACCAGGTAAGAATGAGT	28	35.71	60.69
nCoV-2019_32_LEFT	nCoV-2019_2	TGGTGAATACAGTCATGTAGTTGCC	25	44	61.09
nCoV-2019_32_RIGHT	nCoV-2019_2	AGCACATCACTACGCAACTTTAGA	24	41.67	60.56
nCoV-2019_33_LEFT	nCoV-2019_1	ACTTTTGAAGAAGCTGCGCTGT	22	45.45	61.58
nCoV-2019_33_RIGHT	nCoV-2019_1	TGGACAGTAAACTACGTCATCAAGC	25	44	61.08
nCoV-2019_34_LEFT	nCoV-2019_2	TCCCATCTGGTAAAGTTGAGGGT	23	47.83	61.02
nCoV-2019_34_RIGHT	nCoV-2019_2	AGTGAAATTGGGCCTCATAGCA	22	45.45	60.03
nCoV-2019_35_LEFT	nCoV-2019_1	TGTTCGCATTCAACCAGGACAG	22	50	61.39
nCoV-2019_35_RIGHT	nCoV-2019_1	ACTTCATAGCCACAAGGTTAAAGTCA	26	38.46	60.69
nCoV-2019_36_LEFT	nCoV-2019_2	TTAGCTTGGTTGTACGCTGCTG	22	50	61.44
nCoV-2019_36_RIGHT	nCoV-2019_2	GAACAAAGACCATTGAGTACTCTGGA	26	42.31	60.74
nCoV-2019_37_LEFT	nCoV-2019_1	ACACACCACTGGTTGTTACTCAC	23	47.83	60.93
nCoV-2019_37_RIGHT	nCoV-2019_1	GTCCACACTCTCCTAGCACCAT	22	54.55	61.48
nCoV-2019_38_LEFT	nCoV-2019_2	ACTGTGTTATGTATGCATCAGCTGT	25	40	60.86
nCoV-2019_38_RIGHT	nCoV-2019_2	CACCAAGAGTCAGTCTAAAGTAGCG	25	48	61.13
nCoV-2019_39_LEFT	nCoV-2019_1	AGTATTGCCCTATTTTCTTCATAACTGGT	29	34.48	61
nCoV-2019_39_RIGHT	nCoV-2019_1	TGTAACTGGACACATTGAGCCC	22	50	60.55
nCoV-2019_40_LEFT	nCoV-2019_2	TGCACATCAGTAGTCTTACTCTCAGT	26	42.31	61.25
nCoV-2019_40_RIGHT	nCoV-2019_2	CATGGCTGCATCACGGTCAAAT	22	50	62.09
nCoV-2019_41_LEFT	nCoV-2019_1	GTTCCCTTCCATCATATGCAGCT	23	47.83	60.75
nCoV-2019_41_RIGHT	nCoV-2019_1	TGGTATGACAACCATTAGTTTGGCT	25	40	60.75
nCoV-2019_42_LEFT	nCoV-2019_2	TGCAAGAGATGGTTGTGTTCCC	22	50	61.08

nCoV-2019_42_RIGHT	nCoV-2019_2	CCTACCTCCCTTTGTTGTGTTGT	23	47.83	60.69
nCoV-2019_43_LEFT	nCoV-2019_1	TACGACAGATGTCTTGTGCTGC	22	50	60.93
nCoV-2019_43_RIGHT	nCoV-2019_1	AGCAGCATCTACAGCAAAAGCA	22	45.45	61.14
nCoV-2019_44_LEFT	nCoV-2019_2	TGCCACAGTACGTCTACAAGCT	22	50	61.66
nCoV-2019_44_LEFT_alt3	nCoV-2019_2	CCACAGTACGTCTACAAGCTGG	22	54.55	60.67
nCoV-2019_44_RIGHT	nCoV-2019_2	AACCTTTCCACATACCGCAGAC	22	50	60.87
nCoV-2019_44_RIGHT_alt0	nCoV-2019_2	CGCAGACGGTACAGACTGTGTT	22	54.55	62.77
nCoV-2019_45_LEFT	nCoV-2019_1	TACCTACAACTTGTGCTAATGACCC	25	44	60.57
nCoV-2019_45_LEFT_alt2	nCoV-2019_1	AGTATGTACAAATACCTACAACTTGTGCT	29	34.48	60.94
nCoV-2019_45_RIGHT	nCoV-2019_1	AAATTGTTTCTTCATGTTGGTAGTTAGAGA	30	30	60.01
nCoV-2019_45_RIGHT_alt7	nCoV-2019_1	TTCATGTTGGTAGTTAGAGAAAGTGTGTC	29	37.93	61.53
nCoV-2019_46_LEFT	nCoV-2019_2	TGTCGCTTCCAAGAAAAGGACG	22	50	61.38
nCoV-2019_46_LEFT_alt1	nCoV-2019_2	CGCTTCCAAGAAAAGGACGAAGA	23	47.83	61.35
nCoV-2019_46_RIGHT	nCoV-2019_2	CACGTTCACCTAAGTTGGCGTA	22	50	60.86
nCoV-2019_46_RIGHT_alt2	nCoV-2019_2	CACGTTCACCTAAGTTGGCGTAT	23	47.83	61.17
nCoV-2019_47_LEFT	nCoV-2019_1	AGGACTGGTATGATTTTGTAGAAAACCC	28	39.29	61.42
nCoV-2019_47_RIGHT	nCoV-2019_1	AATAACGGTCAAAGAGTTTTAACCTCTC	28	35.71	60.06
nCoV-2019_48_LEFT	nCoV-2019_2	TGTTGACACTGACTTAACAAAGCCT	25	40	61.09
nCoV-2019_48_RIGHT	nCoV-2019_2	TAGATTACCAGAAGCAGCGTGC	22	50	60.74
nCoV-2019_49_LEFT	nCoV-2019_1	AGGAATTACTTGTGTATGCTGCTGA	25	40	60.57
nCoV-2019_49_RIGHT	nCoV-2019_1	TGACGATGACTTGGTTAGCATTAATACA	28	35.71	61.05
nCoV-2019_50_LEFT	nCoV-2019_2	GTTGATAAGTACTTTGATTGTTACGATGGT	30	33.33	60.59
nCoV-2019_50_RIGHT	nCoV-2019_2	TAACATGTTGTGCCAACCACCA	22	45.45	60.95
nCoV-2019_51_LEFT	nCoV-2019_1	TCAATAGCCGCCACTAGAGGAG	22	54.55	61.34
nCoV-2019_51_RIGHT	nCoV-2019_1	AGTGCATTAACATTGGCCGTGA	22	45.45	61.14
nCoV-2019_52_LEFT	nCoV-2019_2	CATCAGGAGATGCCACAACTGC	22	54.55	61.83
nCoV-2019_52_RIGHT	nCoV-2019_2	GTTGAGAGCAAAATTCATGAGGTCC	25	44	60.62
nCoV-2019_53_LEFT	nCoV-2019_1	AGCAAAATGTTGGACTGAGACTGA	24	41.67	60.69
nCoV-2019_53_RIGHT	nCoV-2019_1	AGCCTCATAAAACTCAGGTTCCC	23	47.83	60.31
nCoV-2019_54_LEFT	nCoV-2019_2	TGAGTTAACAGGACACATGTTAGACA	26	38.46	60.18
nCoV-2019_54_RIGHT	nCoV-2019_2	AACCAAAAACTTGTCCATTAGCACA	25	36	60.11
nCoV-2019_55_LEFT	nCoV-2019_1	ACTCAACTTTACTTAGGAGGTATGAGCT	28	39.29	61.43

nCoV-2019_55_RIGHT	nCoV-2019_1	GGTGTACTCTCCTATTTGTACTTTACTGT	29	37.93	60.54
nCoV-2019_56_LEFT	nCoV-2019_2	ACCTAGACCACCACTTAACCGA	22	50	60.49
nCoV-2019_56_RIGHT	nCoV-2019_2	ACACTATGCGAGCAGAAGGGTA	22	50	61.21
nCoV-2019_57_LEFT	nCoV-2019_1	ATTCTACACTCCAGGGACCACC	22	54.55	61.16
nCoV-2019_57_RIGHT	nCoV-2019_1	GTAATTGAGCAGGGTCGCCAAT	22	50	61.26
nCoV-2019_58_LEFT	nCoV-2019_2	TGATTTGAGTGTTGTCAATGCCAGA	25	40	61.44
nCoV-2019_58_RIGHT	nCoV-2019_2	CTTTTCTCCAAGCAGGGTTACGT	23	47.83	61.06
nCoV-2019_59_LEFT	nCoV-2019_1	TCACGCATGATGTTTCATCTGCA	23	43.48	61.42
nCoV-2019_59_RIGHT	nCoV-2019_1	AAGAGTCCTGTTACATTTTCAGCTTG	26	38.46	60.02
nCoV-2019_60_LEFT	nCoV-2019_2	TGATAGAGACCTTTATGACAAGTTGCA	27	37.04	60.53
nCoV-2019_60_RIGHT	nCoV-2019_2	GGTACCAACAGCTTCTCTAGTAGC	24	50	60.44
nCoV-2019_61_LEFT	nCoV-2019_1	TGTTTATCACCCGCGAAGAAGC	22	50	61.5
nCoV-2019_61_RIGHT	nCoV-2019_1	ATCACATAGACAACAGGTGCGC	22	50	61.25
nCoV-2019_62_LEFT	nCoV-2019_2	GGCACATGGCTTTGAGTTGACA	22	50	61.91
nCoV-2019_62_RIGHT	nCoV-2019_2	GTTGAACCTTTCTACAAGCCGC	22	50	60.35
nCoV-2019_63_LEFT	nCoV-2019_1	TGTTAAGCGTGTTGACTGGACT	22	45.45	60.16
nCoV-2019_63_RIGHT	nCoV-2019_1	ACAAACTGCCACCATCACAACC	22	50	61.85
nCoV-2019_64_LEFT	nCoV-2019_2	TCGATAGATATCCTGCTAATTCCATTGT	28	35.71	60.11
nCoV-2019_64_RIGHT	nCoV-2019_2	AGTCTTGTAAAAGTGTTCCAGAGGT	25	40	60.1
nCoV-2019_65_LEFT	nCoV-2019_1	GCTGGCTTTAGCTTGTGGGTTT	22	50	61.92
nCoV-2019_65_RIGHT	nCoV-2019_1	TGTCAGTCATAGAACAAACACCAATAGT	28	35.71	60.9
nCoV-2019_66_LEFT	nCoV-2019_2	GGGTGTGGACATTGCTGCTAAT	22	50	61.21
nCoV-2019_66_RIGHT	nCoV-2019_2	TCAATTTCCATTTGACTCCTGGGT	24	41.67	60.45
nCoV-2019_67_LEFT	nCoV-2019_1	GTTGTCCAACAATTACCTGAAACTTACT	28	35.71	60.43
nCoV-2019_67_RIGHT	nCoV-2019_1	CAACCTTAGAAACTACAGATAAATCTTGGG	30	36.67	60.4
nCoV-2019_68_LEFT	nCoV-2019_2	ACAGGTTCATCTAAGTGTGTGTGT	24	41.67	60.14
nCoV-2019_68_RIGHT	nCoV-2019_2	CTCCTTTATCAGAACCAGCACCA	23	47.83	60.31
nCoV-2019_69_LEFT	nCoV-2019_1	TGTCGCAAAATATACTCAACTGTGTCA	27	37.04	61.43
nCoV-2019_69_RIGHT	nCoV-2019_1	TCTTTATAGCCACGGAACCTCCA	23	47.83	61.14
nCoV-2019_70_LEFT	nCoV-2019_2	ACAAAAGAAAATGACTCTAAAGAGGGTTT	29	31.03	60.13
nCoV-2019_70_RIGHT	nCoV-2019_2	TGACCTTCTTTTAAAGACATAACAGCAG	28	35.71	60.27
nCoV-2019_71_LEFT	nCoV-2019_1	ACAAATCCAATTCAGTTGTCTTCCTATTC	29	34.48	60.54

nCoV-2019_71_RIGHT	nCoV-2019_1	TGGAAAAGAAAGGTAAGAACAAGTCCT	27	37.04	60.8
nCoV-2019_72_LEFT	nCoV-2019_2	ACACGTGGTGTTTATTACCCTGAC	24	45.83	61.04
nCoV-2019_72_RIGHT	nCoV-2019_2	ACTCTGAACTCACTTTCCATCCAAC	25	44	60.97
nCoV-2019_73_LEFT	nCoV-2019_1	CAATTTTGTAATGATCCATTTTTGGGTGT	29	31.03	60.29
nCoV-2019_73_RIGHT	nCoV-2019_1	CACCAGCTGTCCAACCTGAAGA	22	54.55	62.45
nCoV-2019_74_LEFT	nCoV-2019_2	ACATCACTAGGTTTCAAACTTTACTTGC	28	35.71	60.68
nCoV-2019_74_RIGHT	nCoV-2019_2	GCAACACAGTTGCTGATTCTCTTC	24	45.83	60.85
nCoV-2019_75_LEFT	nCoV-2019_1	AGAGTCCAACCAACAGAATCTATTGT	26	38.46	60.24
nCoV-2019_75_RIGHT	nCoV-2019_1	ACCACCAACCTTAGAATCAAGATTGT	26	38.46	60.69
nCoV-2019_76_LEFT	nCoV-2019_2	AGGGCAAACTGGAAAGATTGCT	22	45.45	60.76
nCoV-2019_76_LEFT_alt3	nCoV-2019_2	GGGCAAACTGGAAAGATTGCTGA	23	47.83	61.87
nCoV-2019_76_RIGHT	nCoV-2019_2	ACACCTGTGCCTGTTAAACCAT	22	45.45	60.42
nCoV-2019_76_RIGHT_alt0	nCoV-2019_2	ACCTGTGCCTGTTAAACCATTGA	23	43.48	60.69
nCoV-2019_77_LEFT	nCoV-2019_1	CCAGCAACTGTTTGTGGACCTA	22	50	60.75
nCoV-2019_77_RIGHT	nCoV-2019_1	CAGCCCCTATTAAACAGCCTGC	22	54.55	61.59
nCoV-2019_78_LEFT	nCoV-2019_2	CAACTTACTCCTACTTGGCGTGT	23	47.83	60.55
nCoV-2019_78_RIGHT	nCoV-2019_2	TGTGTACAAAAACTGCCATATTGCA	25	36	60.22
nCoV-2019_79_LEFT	nCoV-2019_1	GTGGTGATTCAACTGAATGCAGC	23	47.83	60.92
nCoV-2019_79_RIGHT	nCoV-2019_1	CATTTCATCTGTGAGCAAAGGTGG	24	45.83	60.62
nCoV-2019_80_LEFT	nCoV-2019_2	TTGCCTTGGTGATATTGCTGCT	22	45.45	60.89
nCoV-2019_80_RIGHT	nCoV-2019_2	TGGAGCTAAGTTGTTTAACAAGCG	24	41.67	60.02
nCoV-2019_81_LEFT	nCoV-2019_1	GCACTTGGAAAACTTCAAGATGTGG	25	44	61.24
nCoV-2019_81_RIGHT	nCoV-2019_1	GTGAAGTTCTTTTCTTGTGCAGGG	24	45.83	60.73
nCoV-2019_82_LEFT	nCoV-2019_2	GGGCTATCATCTTATGTCCTTCCCT	25	48	61.52
nCoV-2019_82_RIGHT	nCoV-2019_2	TGCCAGAGATGTCACCTAAATCAA	24	41.67	60.02
nCoV-2019_83_LEFT	nCoV-2019_1	TCCTTTGCAACCTGAATTAGACTCA	25	40	60.46
nCoV-2019_83_RIGHT	nCoV-2019_1	TTTGACTCCTTTGAGCACTGGC	22	50	61.33
nCoV-2019_84_LEFT	nCoV-2019_2	TGCTGTAGTTGTCTCAAGGGCT	22	50	61.61
nCoV-2019_84_RIGHT	nCoV-2019_2	AGGTGTGAGTAAACTGTTACAAACAAC	27	37.04	60.36
nCoV-2019_85_LEFT	nCoV-2019_1	ACTAGCACTCTCCAAGGGTGTT	22	50	61.03
nCoV-2019_85_RIGHT	nCoV-2019_1	ACACAGTCTTTTACTCCAGATTCCC	25	44	60.51
nCoV-2019_86_LEFT	nCoV-2019_2	TCAGGTGATGGCACAACAAGTC	22	50	61.07

nCoV-2019_86_RIGHT	nCoV-2019_2	ACGAAAGCAAGAAAAAGAAGTACGC	25	40	61.01
nCoV-2019_87_LEFT	nCoV-2019_1	CGACTACTAGCGTGCCTTTGTA	22	50	60.16
nCoV-2019_87_RIGHT	nCoV-2019_1	ACTAGGTTCCATTGTTCAAGGAGC	24	45.83	60.81
nCoV-2019_88_LEFT	nCoV-2019_2	CCATGGCAGATTCCAACGGTAC	22	54.55	61.58
nCoV-2019_88_RIGHT	nCoV-2019_2	TGGTCAGAATAGTGCCATGGAGT	23	47.83	61.4
nCoV-2019_89_LEFT	nCoV-2019_1	GTACGCGTTCCATGTGGTCATT	22	50	61.5
nCoV-2019_89_LEFT_alt2	nCoV-2019_1	CGCGTTCCATGTGGTCATTCAA	22	50	62.01
nCoV-2019_89_RIGHT	nCoV-2019_1	ACCTGAAAGTCAACGAGATGAAACA	25	40	60.91
nCoV-2019_89_RIGHT_alt4	nCoV-2019_1	ACGAGATGAAACATCTGTTGTCACT	25	40	60.74
nCoV-2019_90_LEFT	nCoV-2019_2	ACACAGACCATTCCAGTAGCAGT	23	47.83	61.58
nCoV-2019_90_RIGHT	nCoV-2019_2	TGAAATGGTGAATTGCCCTCGT	22	45.45	60.82
nCoV-2019_91_LEFT	nCoV-2019_1	TCACTACCAAGAGTGTGTTAGAGGT	25	44	60.93
nCoV-2019_91_RIGHT	nCoV-2019_1	TTCAAGTGAGAACCAAAAGATAATAAGCA	29	31.03	60.03
nCoV-2019_92_LEFT	nCoV-2019_2	TTTGTGCTTTTTAGCCTTTCTGCT	24	37.5	60.14
nCoV-2019_92_RIGHT	nCoV-2019_2	AGGTTCCTGGCAATTAATTGTAAAAGG	27	37.04	60.53
nCoV-2019_93_LEFT	nCoV-2019_1	TGAGGCTGGTTCTAAATCACCCA	23	47.83	61.59
nCoV-2019_93_RIGHT	nCoV-2019_1	AGGTCTTCCTTGCCATGTTGAG	22	50	60.55
nCoV-2019_94_LEFT	nCoV-2019_2	GGCCCCAAGGTTTACCCAATAA	22	50	60.56
nCoV-2019_94_RIGHT	nCoV-2019_2	TTTGGCAATGTTGTTCCTTGAGG	23	43.48	60.18
nCoV-2019_95_LEFT	nCoV-2019_1	TGAGGGAGCCTTGAATACACCA	22	50	61.1
nCoV-2019_95_RIGHT	nCoV-2019_1	CAGTACGTTTTTGCCGAGGCTT	22	50	61.95
nCoV-2019_96_LEFT	nCoV-2019_2	GCCAACAACAACAAGGCCAAAC	22	50	61.82
nCoV-2019_96_RIGHT	nCoV-2019_2	TAGGCTCTGTTGGTGGGAATGT	22	50	61.36
nCoV-2019_97_LEFT	nCoV-2019_1	TGGATGACAAAGATCCAAATTTCAAAGA	28	32.14	60.22
nCoV-2019_97_RIGHT	nCoV-2019_1	ACACACTGATTAAAGATTGCTATGTGAG	28	35.71	60.17
nCoV-2019_98_LEFT	nCoV-2019_2	AACAATTGCAACAATCCATGAGCA	24	37.5	60.5
nCoV-2019_98_RIGHT	nCoV-2019_2	TTCTCCTAAGAAGCTATTAAAATCACATGG	30	33.33	60.01
