# Supplementary material for: Transmission of SARS-CoV-2 in domestic cats imposes a narrow bottleneck
Source: PLoS Pathog. 2021 Feb 26;17(2):e1009373. doi: 10.1371/journal.ppat.1009373 (PMC7946358; doi:10.1371/journal.ppat.1009373)
Supplement: Attachment — Submitted filename: Response to reviewer comments.pdf [file ppat.1009373.s016.pdf]

Text which has been edited/added in the manuscript is highlighted in yellow.
Please note that line numbers included here correspond to the revised manuscript unless otherwise specified.

Reviewer #1

1. *Braun et al have examined the transmission bottleneck of SARS-CoV-2 in domestic co-housed cats. In a serious of well-defined analyses, the authors define the SNV in donor and recipient animals to quantify the bottleneck and extrapolate that 2-5 infectious viruses likely seeds subsequent infections. In 2 of the 3 pairs of cats, the authors describe a potential selective advantage of Spike mutation H665Y and Envelope protein mutation S67S. The H665Y mutation has emerged in the human population and it interesting that is would in cats as well. However, this mutation was not observed in any of the deposited feline SARS-CoV-2 sequences, which is surprising.*

   Overall the authors provide a clear explanation of the bottleneck analysis and the discussion describes the nuance associated with these types of analyses. The bottleneck size of close contact transmission is important to understand as the authors demonstrate may be driven by a very small dose of virus particles. We provide a few suggestions that could increase the clarity and enhance the biological insight of this manuscript, but overall this article is of broad interest and timely.

   We thank the reviewer for these encouraging comments.

2. *The authors reference the robust replication of SARS-CoV-2 in domestic cats in multiple anatomical sites (ref 1, Shi et al). Are the authors aware of whether the H665Y mutation emerged in all anatomical sites, or is there a preference for the upper respiratory tract (given the site of nasal wash). The prevalence in other organs may influence the amount of this variant released into environment and could alter some of the bottleneck calculations. If that data are not available – perhaps a mention of how variations of viral adaptation within anatomical sites, could influence bottleneck calculations in the discussion is warranted.*

   We agree the anatomic location of variant emergence as well as the concept of independently replicating foci of infection are important and interesting concepts. Domestic cats involved in these experiments were not sacrificed following the acute infection so while characterizing SARS-CoV-2 genetic diversity across anatomic compartments is of interest to us, we only have access to nasal swabs and can therefore only investigate diversity in the upper respiratory tract. Per the reviewers’

excellent suggestion, we have added a few sentences on this concept to the discussion (lines 342-349).

3. *The emergence of H665Y variants in Spike has caused a great deal of concern given the escape form certain monoclonal antibodies. It would be interesting if the authors could use the cat sera from donors (where is this variant is low) to demonstrate neutralization of viruses carrying this mutation. Data such as this could help address whether polyclonal responses, particular during natural infection would still recognize this new variant. The corresponding study describing the cat transmission experiments (ref 33, Halfmann et al), only specifies ELISA antibody titers, but neutralizing titers – may be more informative. However, this aspect may be beyond the scope of this manuscript.*

   We thank the reviewer for this comment and we agree these experiments would be useful. Unfortunately they are indeed beyond the scope of this current manuscript.

Reviewer #2

1. *In the present study by Braun and colleagues titled "Transmission of SARS-CoV-2 in domestic cats imposes a narrow bottleneck", SARS-CoV-2 genetic diversity is assessed within and between hosts using the COVID-19 cat transmission model. The authors determined how SARS-CoV-2 might evolve as it continues to spread to various susceptible animal species. They could show that narrow transmission bottlenecks and genetic drift are the major forces shaping SARS-CoV-2 genetic diversity in cats. The results also suggest that a positive selective pressure may exist for the H655Y spike variant which was identified in all index cats; this spike variant is also described in other experimental animal models, tissue culture studies and in the human population. The authors conclude that the COVID-19 cat transmission model recapitulates key aspects of SARS-CoV-2 evolution in humans. In the cat model, within host genetic variation is predominantly influenced by genetic drift and purifying selection and transmission between cats is defined by a narrow transmission bottleneck involving only 2-5 viruses. Therefore, the COVID-19 cat model might serve as a model system to study SARS-CoV-2 diversity and evolution within and between hosts.*

   The manuscript is well written and contributes to our understanding of SARS-CoV-2 diversity and evolution. The introduction provides sufficient background and the conclusions appear to be supported by the data. The results are presented and illustrated clearly by the figures and discussed appropriately. The analysis appears to be thorough and the methods and statistics used appear appropriate. The identification of the SARS-CoV-2 variant H655Y being positively selected in cats is interesting. Since this variant was previously shown escape from monoclonal antibodies and the substitution is

close to the fusion peptide, the authors hypothesize that this site in the spike protein may be under positive selection in cats and being potentially beneficial for cat replication. In this context, it is important to know whether the cats used in the experiment were seronegative not only for SARS-CoV-2-specific antibodies but also for antibodies against feline coronaviruses. In addition, data using H655Y pseudoviruses or plaque-purified/rescued H655Y variant viruses should be presented to help answering questions regarding the functional impact (e.g., fusion efficiency, host entry; immune escape) of the H655Y substitution. The question still remains why only two out of three contact cats selected the H655Y variant.

We thank the reviewer for this accurate summary of our study and thoughtful comments regarding important next experimental steps.

We agree the cats' serostatus against SARS-CoV-2 and other coronaviruses is important here to accurately interpret the emergence of Spike H655Y. All index and contact cats had undetectable IgG antibodies against SARS-CoV-2 Spike receptor binding domain and nucleocapsid protein by ELISA. This assay is cross-reactive to feline coronaviruses. Further, these cats were maintained in a specific pathogen free (SPF) colony. We address this briefly in the discussion on lines 297-300 (original submission line numbering). We further clarify this point in the new methods section on lines 383-394.

While we agree that experiments aimed to characterize the phenotypic and functional impacts of Spike H655Y in SARS-CoV-2 in cats and in humans are needed, we are not positioned to perform these experiments in a timely manner and believe these experiments are outside the scope of this manuscript. We have included a new supplementary figure, **Supplementary Figure 12**, to illustrate the physical location of Spike H655Y relative to the Spike glycoprotein trimer and the human ACE2 receptor (generated using GISAID's CoVsurver). We additionally include summary information regarding the impact of a histidine to tyrosine change on charge, volume, and aromaticity.

2. *Reviewer #2: Lines 268-271: What about comorbidities? Please clarify.*

   Thank you for pointing out this oversight. We have added relevant comorbidities on lines 268-271.

3. *Figure 3: Please add respective cat numbers to the Figure.*

   Thank you for this suggestion. We have added the cat numbers to Figure 3.

4. *Figure 4. Define Nb.*

   "Nb" is a common abbreviation for bottleneck size, or the estimated number of viruses founding productive infection in the contact cat. We defined this on the y-axis of the right subplot in this figure. However, its inclusion is not necessary here so we have removed "Nb" from this figure for clarity.

5. *Suppl. Figure 3: It is very difficult to read this figure. Please improve coloring, etc.*

   We have done our best to improve the readability of supplementary figure 3. Most indels are very low frequency and so individual indels are overlapping, making it difficult to see individual variants. We adjusted the y-axis range so the individual indels are more easily seen now. We note the shifted y-axis range in the figure legend.
